# Allergen‐Specific Immune Responses‐Sex Matters

**DOI:** 10.1002/clt2.70222

**Published:** 2026-11-15

**Authors:** Alisa Landgraf, Angelina Eisenhauer, Sascha Döring, Daniel Strecker, Michelle Beatrice Wolff, Ann‐Christine Junker, Meike Arend, Kathrin Elisabeth Paulus‐Tremel, Sandra Schmidt, Susanne Kaul, Stefan Schülke, Vera Mahler

**Affiliations:** ^1^ Allergology Division Paul‐Ehrlich‐Institut Langen Germany; ^2^ Friedrich‐Alexander‐Universität Erlangen‐Nürnberg (FAU) Erlangen Germany

**Keywords:** allergen immunotherapy (AIT), Api *m* 10, honey bee venom allergy, icarapin, sex‐specific immune responses, venom immunotherapy (VIT)

**Abbreviations**
AIT: allergen‐specific immunotherapy
Api *m* 10: *Apis mellifera* allergen 10, icarapin
HBV: honey bee venom
OD: optical density

To the editor,

Wasp venom and honey bee venom (HBV) cause severe venom allergies in humans [1]. As disease‐modifying treatment allergen‐specific immunotherapy (AIT) with authorized medicinal products manufactured from natural HBV is available. These AIT‐products are administered according to fixed dosage schemes irrespective of sex or body weight of patients. It is currently unknown whether these factors influence therapy outcome and studies analyzing weight‐ and sex differences during either allergic sensitization or AIT are scarce [2].

In a retrospective study, sensitization to Api *m* 10, a major HBV‐allergen, was suggested as predictor for therapy failure [3]. Therefore, insights into Api *m* 10‐induced immune responses may improve the treatment of HBV‐allergy.

Clinical differences between female and male patients have been described for several allergies [4]. In the present project, we studied whether humoral immune responses induced by Api *m* 10 differ in female and male mice. For this, age‐matched female and male BALB/c were immunized with increasing amounts of recombinant Api *m* 10 in 14‐day intervals (see supplement for material and methods). Induction of Api *m* 10‐specific pan‐IgG‐production was monitored and immunizations were continued until the pre‐defined endpoint was reached (OD_450_ ≥ 1 of Api *m* 10‐specific pan‐IgG in indirect ELISA at serum dilution of 1–10,000; Figure 1A). Upon repeated immunization with increasing amounts of Api *m* 10, female mice developed Api *m* 10‐specific pan‐IgG antibodies significantly earlier than male mice (Figure 1B). Antibody response trajectories differed significantly between the two groups over time (*p* < 0.001) with significant differences between female and male animals observed from day 98 onwards (day 98: *p* = 0.0134, day 112: *p* < 0.001, day 126: *p* < 0.001, day 140: *p* < 0.001; Figure 1B). At the end of the experiment, male mice still showed significantly lower levels of Api *m* 10‐specific IgG_2a_, IgG_2b_, and IgE antibodies (Figure 1C).

**FIGURE 1:**
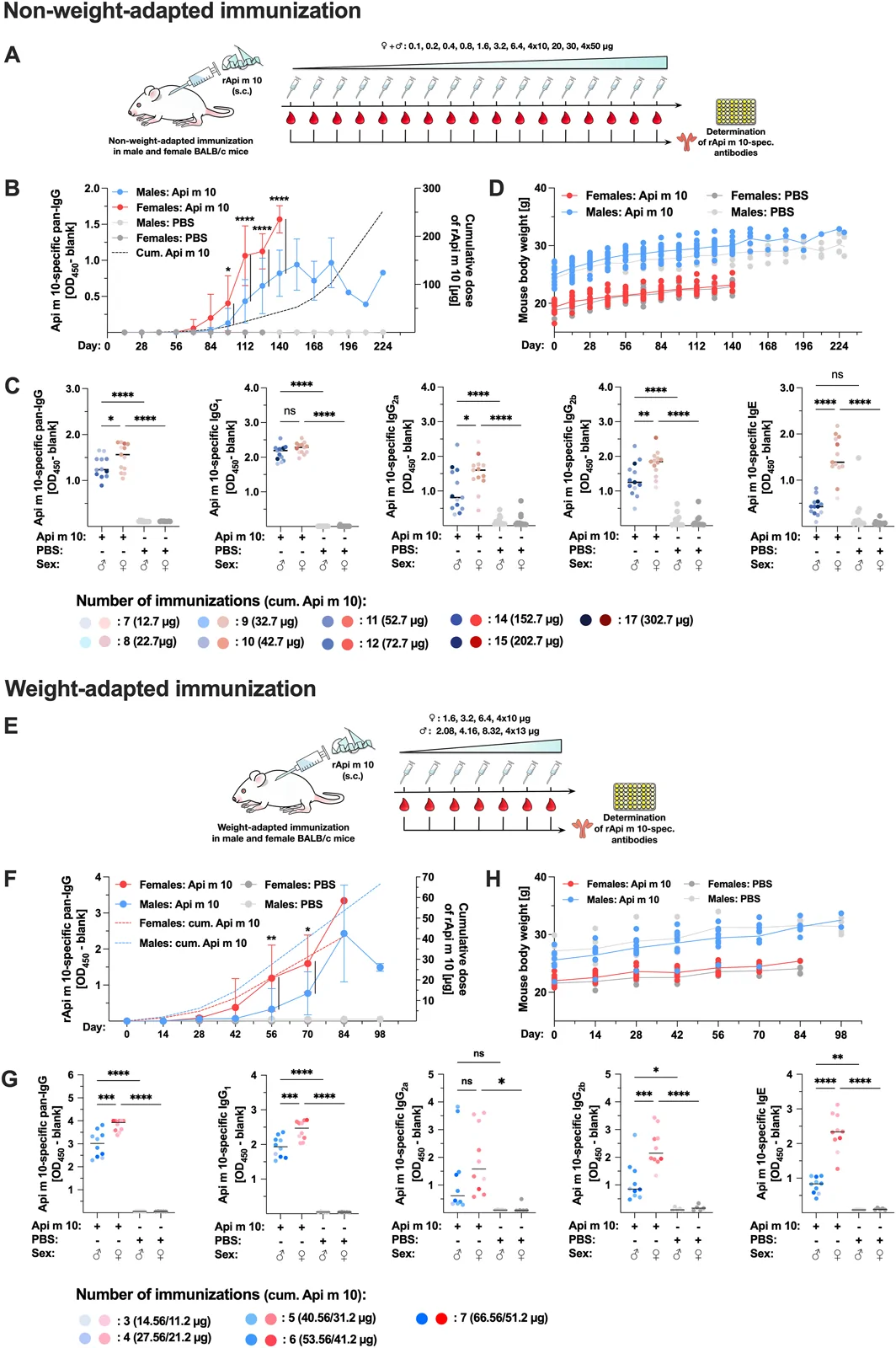
Antibody responses to the major honey bee venom allergen Api *m* 10 differ in male and female mice. (A) Male and female BALB/c mice were immunized either nonweight‐adapted (A–D) or weight‐adapted (E–H) with Api *m* 10 until reaching the pre‐defined endpoint. (B and F) Time course of Api m 10‐specific pan‐IgG production. (C and G) Api *m* 10‐specific antibody isotype levels in sera upon reaching the pre‐defined endpoint. (D and H) Time course of mouse body weight. Graphs based on either (I) 13 (Api *m* 10) or 12 (PBS) animals per group (B–D) or (II) 10 (Api *m* 10) or 5 (PBS) animals per group (F–H).

The two most likely reasons for the observed sex‐differences in antibody production, that could be studied, are differences in body weight and hormonal differences. Indeed, throughout the experiment, male mice were approx. 30% heavier than age‐matched female mice (significant differences in body weight were observed between male and female mice with *p* > 0.0001 for time points day 0–140, Figure 1D).

To investigate whether the observed differences in Api *m* 10‐specific antibody responses were due to differences in total body weight, the study was repeated with a weight‐adapted study protocol where male mice—in line with the previously observed weight differences—on a group level received 30% higher Api *m* 10‐doses than their female counterparts (for details: see Supporting Information S1; Figure 1E).

Also upon weight‐adjusted immunization, female mice developed Api *m* 10‐specific pan‐IgG antibodies faster with antibody response trajectories differing significantly over time between Api *m* 10‐immunized male and female mice (*p* < 0.0228, Figure 1F). Significant differences within specific timepoints were observed on days 56 (*p* = 0.0037) and 70 (*p* < 0.03, Figure 1F). Additionally, female mice showed significantly higher pan‐IgG levels than male mice at the end of the experiment despite receiving lower cumulative amounts of Api *m* 10 (Figure 1F and G). Furthermore, at the end of the experiment, sera from female mice contained significantly higher amounts of Api *m* 10‐specific IgG_1_, IgG_2b_, and IgE (Figure 1G). In line with the first study (Figure 1A–C) male mice showed an approx. 30% higher body weight (significant differences in body weight were observed between male and female mice with *p* > 0.0001 for time points day 0–84, Figure 1H).

Api *m* 10‐specific pan‐IgG ratios between sexes were comparable between both studies (nonweight‐adjusted immunization: 1.27‐fold more pan‐IgG in females than in males, weight adjusted immunization: 1.30‐fold more pan‐IgG in females than in males), whereas Api *m* 10‐specific subtype ratios between female and male animals were increased upon weight‐adjusted immunization (nonweight‐adjusted vs. weight‐adjusted immunization: IgG_1_: 1.03‐fold vs. 1.28‐fold; IgG_2a_: 1.98‐fold vs. 2.59‐fold; IgG_2b_: 1.53‐fold vs. 2.53‐fold). Api *m* 10‐specific IgE ratios were decreased (nonweight‐adjusted: 3.29‐fold vs. weight‐adjusted: 2.81‐fold more IgE in females than in males).

In summary, our results demonstrate that male mice require higher numbers of injections and consequently an increased cumulative Api *m* 10‐dosage compared to their female counterparts to produce allergen‐specific IgG_1_, IgG_2b_, and IgE antibodies. These differences were still observed using a weight‐adjusted immunization scheme, thus suggesting other factors (like hormonal differences) to contribute to this effect. Here, further studies are necessary to explore the contribution of different hormones (i.e., by gonadectomy or hormone replacement therapy). Furthermore, as body weight differences between male and female mice primarily reflect differences in fat‐ and muscle tissue rather than lymphoid tissue, further studies are necessary to clarify the reasons for our observations and their potential consequences for AIT in mouse models and, if applicable, in humans.

Of note, in both studies, female mice produced significantly more Api *m* 10‐specific IgE than male mice. In the weight‐adjusted study, raising the allergen dose administered to male mice increased IgE production in male mice, while in direct comparison female mice were subjected to a slower build‐up phase. With regard to a potential transferability of our results in mice to humans, females (displaying lower average body weights and producing higher amounts of IgE when receiving equal doses of venom AIT‐products) might be at an increased risk of systemic reactions during the venom AIT build‐up phase. Here, some studies have indeed suggested female patients to suffer from higher frequencies of severe adverse reactions during venom AIT [5, 6, 7, 8, 9, 10], while other studies could not confirm this trend [11, 12] and reviewed in [13].

Our results of sex‐specific immune responses in mice are in line with previous findings in a mouse model of house dust mite allergy [14] and observations in humans where allergies are more common in female than in male patients [15]. Also before puberty, asthma is more prevalent and severe in boys [16]. After puberty (which increases testosterone levels in males) asthma is more common in females [16]. These sex differences were attributed to multiple factors including, but likely not limited to, sex hormones, gender‐specific differences in behavior, differences in risk consciousness, or drug use [17].

Noteworthy, sex‐related differences in efficacy and adverse reactions initially observed in a subcutaneous immunotherapy study for allergic rhinitis were found to be no longer significant after propensity matching 57,857 study participants [18]. After propensity matching, the only difference was male study participants reporting a poorer perception of early AIT efficacy, which may reduce male‐patient adherence and influence long‐term AIT outcomes [18]. Here, the authors suggested sex‐related hormonal differences to contribute to the difference in efficacy perception [18].

Also, our results suggest that especially the effects of sex hormones may be paramount for the induced immune response and need to be addressed in future studies. Immunologically, estrogen enhances both Th2 cell function and Th2 antibody responses, whereas testosterone functions as general immune‐suppressor [4]. In line with this, estrogen receptors are found on many immune cells including lymphocytes, monocytes, and mast cells [19] which all are critical in establishing and maintaining allergic responses.

Although sex differences in allergic immune responses remain understudied, our findings contribute to the growing body of evidence suggesting that the sex may influence allergic sensitization. Accordingly, sex‐specific dosage schemes of AIT‐products might contribute to an optimized induction of allergen‐specific blocking IgG antibody responses during AIT. Further insights could be retrieved retrospectively and/or prospectively from clinical trials, that is by sex‐specific evaluation of immune response data of Phase II (dose finding) and both immune response and clinical data of Phase III (confirmatory) studies.

## Author Contributions

**Alisa Landgraf:** writing – review and editing, investigation, formal analysis, methodology, data curation, visualization. **Angelina Eisenhauer:** investigation, methodology. **Sascha Döring:** investigation, methodology. **Daniel Strecker:** investigation, methodology. **Michelle Beatrice Wolff:** investigation, methodology. **Ann‐Christine Junker:** investigation, methodology. **Meike Arend:** investigation, methodology. **Kathrin Elisabeth Paulus‐Tremel:** investigation, methodology. **Sandra Schmidt:** investigation, supervision, conceptualization. **Susanne Kaul:** supervision, writing – review and editing, investigation, conceptualization, project administration. **Stefan Schülke:** investigation, supervision, data curation, formal analysis, methodology, visualization, writing – original draft, writing – review and editing, project administration. **Vera Mahler:** conceptualization, writing – original draft, writing – review and editing, methodology, formal analysis, project administration, resources, supervision, data curation.

## Funding

This work has been funded solely by the budget of the Allergology Division, Paul‐Ehrlich‐Institut, Langen (Hesse), Germany.

## Ethics Statement

All animal experiments were performed in accordance with German animal protection law (*Regierungspräsidium Darmstadt*, approval number: F017/1064).

## Conflicts of Interest

The authors declare no conflicts of interest.

## Disclaimer

The views expressed in this manuscript are the personal views of the authors and may not be understood or quoted as being made on behalf of or reflecting the position of the respective national competent authority, the European Medicines Agency, or one of its committees or working parties.

## Supporting information


Supporting Information S1

## Data Availability

The data that support the findings of this study are available from the corresponding author upon reasonable request.
