# Supplementary material for: Allergen‐Specific Immune Responses‐Sex Matters
Source: Clin Transl Allergy. 2026 Nov 15;16(11):e70222. doi: 10.1002/clt2.70222 (PMC13770541; doi:10.1002/clt2.70222)
Supplement: Supplementary file 1 [file CLT2-16-e70222-s001.docx]

**Allergen-specific immune responses - sex matters**

Alisa Landgraf^1,2^, Angelina Eisenhauer BSc.^1^, Sascha Döring^1^, Daniel Strecker^1^, Michelle Beatrice Wolff MSc.^1^, Ann-Christine Junker MSc.^1^, Meike Arend MSc.^1^, Kathrin Elisabeth Paulus-Tremel PhD^1,3^, Sandra Schmidt PhD^1^, Susanne Kaul DVM^1^, Stefan Schülke Prof. (apl.)^1^, Vera Mahler Prof.^1,2^

^1^ Division of Allergology, Paul-Ehrlich-Institut, Langen, Germany

^2^ Friedrich-Alexander-Universität Erlangen-Nürnberg (FAU), Germany

^3^ present address: Faculty of Life Sciences: Food, Nutrition and Health, University Bayreuth, Kulmbach, Germany

***Correspondence:**

Prof. (apl.) Dr. Stefan Schülke

Phone: +49 6103 775209

Fax: +49 6103 77775209

E-Mail: [Stefan.Schuelke@pei.de](mailto:Vera.Mahler@pei.de)

**Online Repository**

**Material and Methods**

**Immunizations with Api m 10**

Recombinant Api m 10 was generated according to^1^ by overexpression of full-length Api m 10 variant 1 without the signal peptide in *E. coli*. 6- to 8-week-old female and male BALB/c mice (Charles River Laboratories, Sulzfeld, Germany) were immunized with increasing doses of either Api m 10 or PBS in 14-day intervals. For the first (non-weight-adapted) study, 13 female and 13 male mice were immunized with Api m 10 in parallel with control animals (12 female and 12 male mice) which were immunized with PBS. In the second (weight-adapted) study, 10 female and 10 male mice were immunized with Api m 10 in parallel with control animals (5 female and 5 male mice) immunized with PBS. Exclusion criterium was an apparent sickness of the animals. Therefore, at the beginning of the study, animals were visually inspected for their health status. Animals were subsequently randomized cage-wise into either Api m 10- or PBS control groups, groups were non-blinded. Group sizes for the non-weight-adjusted immunization experiments were set to 12 to 13 animals per treatment group. This should allow to detect a difference in means between the groups of approximately 1.325 times the standard deviation using a two-tailed test at a significance level of α = 0.05 with 80% power (no expected differences regarding this research question were known from the literature). For later weight-adapted immunizations, the results from the non-weight-adapted study were taken into account to reduce group sizes to 10 animals per sex for Api m 10 immunizations and 5 animals each for the PBS controls in accordance with the 3R-principles.

For non-weight adapted immunizations mice of both sexes received 0.1, 0.2, 0.4, 0.8, 1.6, 3.2, 6.4, 4x 10, 20, 30, and 4x 50 µg of recombinant (r)Api m 10. Assuming a mean body weight of 25 g per mouse, this dosage scheme equals 4 µg/kg body weight at lowest dose and 2 mg/kg body weight at highest dose of recombinant (r)Api m 10. For weight-adapted immunization male mice received 30% more Api m 10 than their female counterparts (female mice: 1.6, 3.2, 6.4, and 4x 10 µg Api m 10, male mice: 2.08, 4.16, 8.32, and 4x 13 µg Api m 10). Therefore, adjustment of immunization dosages was done on a group level, rather than as individually weight-adjusted dosages. Higher Api m 10 doses were not applied in this experiment as all animals reached the pre-defined endpoint with the indicated doses. Immunizations were performed in 100 µL PBS per dose subcutaneously (s.c.) using Injekt^®^-F Solo syringes (#720-2561, Braun, VWR, Radnor, PA, USA) and tube SafetyGlide™ needles (16 mm (5/8"), #305901, BD, Franklin Lakes, NJ, USA). Animal weights were determined every two weeks and mice were bled every 14 days from the tail vein (using hollow needles (#4657624, Sterican No.12: 0.70 x 30 mm, B. Braun, Melsungen, Germany) directly before the respective immunizations. Blood samples were centrifuged at 4°C for 20 min and 10.000 rpm and serum was stored at -20°C until ELISAs were performed. All animal experiments were performed in accordance with German animal protection law (*Regierungspräsidium Darmstadt*, approval number: F017/1064).

**ELISA**

Api m 10-specific antibody levels were determined by ELISA according to^2^. Briefly, Api m 10 coated Nunc-Immuno™ F96 well microtiter plates (#735-0199, MaxiSorp™, Thermo Fisher Scientific, Waltham, MA, USA), blocked with 200 µL of 10% fetal calf serum (FCS) in PBS (#F7524, Sigma-Aldrich, St. Louis, MO, USA), were incubated with 50 µL of mouse serum for 2 h at room temperature. Sera were diluted either 1:10,000 (for determination of Api m 10-specifc pan-IgG and -IgG_1_) or 1:1000 (Api m 10-specific IgG_2a_, -IgG_2b_, -IgG_3_, and -IgE). Detection was performed for 1 h with 100 µL of one of the following secondary antibodies coupled with horseradish peroxidase (HRP): goat anti-mouse pan-IgG (γ-chain specific)-HRP antibody (#A3673, Sigma-Aldrich, St. Louis, MO, USA) diluted 1:10,000, goat anti-mouse IgG_1_-HRP (#1071-05, SouthernBiotech, Birmingham, AL, USA) diluted 1:16,000, goat anti-mouse IgG_2a_-HRP (#1081-05, SouthernBiotech, Birmingham, AL, USA) diluted 1:500, goat anti-mouse IgG_2b_-HRP (#1091-05, SouthernBiotech, Birmingham, AL, USA) diluted 1:500, goat anti-mouse IgG_3_-HRP (#1101-05, SouthernBiotech, Birmingham, AL, USA) diluted 1:500, or goat anti-mouse IgE-HRP (#1110-05, SouthernBiotech, Birmingham, AL, USA) diluted 1:500.

Detection was performed with 100 µL of substrate solution (21 mM tetramethylbenzidine (TMB, #860336, Sigma-Aldrich, St. Louis, MO, USA) and 0.03% H_2_O_2_ (#107209, Merck, Darmstadt, Germany) in citrate buffer pH 4.0 (0.23 M citric acid, #X863.2, Carl Roth Laborbedarf, Karlsruhe, Germany) and 0.3 M potassium hydroxide (#1.05021, Merck, Darmstadt, Germany). The reaction was stopped by adding 50 µL of 25% sulfuric acid (#0967.2, Carl Roth Laborbedarf, Karlsruhe, Germany) after either 5 min (for detection of IgG_1_, IgG_2a_, IgG_2b_), 8 min (for IgE), or 15 min (pan-IgG, IgG_3_). Optical densities (OD) were measured at 450 nm using a SpectraMax^®^ABS microplate reader (Molecular Devices, CA, USA).

**Statistical analyses**

Statistical tests were performed with GraphPad Prism v9.5.0 (GraphPad Software, LLC, Boston, MA, USA) using one-way ANOVA with confidence intervals adjusted for multiple comparisons according to Tukey^3^ (Figures 1C and G). Longitudinal antibody responses were analyzed in GraphPad Prism using a two-way mixed-effects model with restricted maximum likelihood (REML). Experimental group and time were included as fixed effects, and the group × time interaction was assessed to determine whether antibody-response trajectories differed between groups over time. Repeated measurements obtained from the same animal were treated as matched observations. Missing measurements resulting from animals reaching the pre-defined endpoint were left as missing and were not imputed. Where applicable, differences between groups at individual time points were assessed using post hoc multiple-comparison tests with appropriate adjustment for multiple testing according to Šidák (Figures 1B, 1D, 1F, and 1H). For statistically significant results the following convention was used: *: p-value < 0.05, **: p-value < 0.01, ***: p-value < 0.001, ****: p-value < 0.0001, ns: not significant;

**References**

1.	Landgraf A, Hein S, Paulus-Tremel KE, et al. Establishment of an ELISA-based in vitro test system to quantify the major honey bee venom allergen Api m 10 in crude bee venoms and therapy allergen products. *Allergy*. 2025;80(3):861-864. doi:10.1111/all.16313

2.	Landgraf A, Paulus-Tremel KE, Zeigermann C, et al. Low Api m 10 in Commercial Honeybee Venom AIT Products Fails to Induce Prominent Humoral Responses in BALB/c Mice. *J Investig Allergol Clin Immunol*. Published online December 11, 2025:0. doi:10.18176/jiaci.1135

3.	Tukey JW. Comparing individual means in the analysis of variance. *Biometrics*. 1949;5(2):99-114.
